# Associations between education and awareness of government services among older adults: an integrated approach to examine the role of digital technology use

**DOI:** 10.1093/geroni/igaf146

**Published:** 2025-12-15

**Authors:** Min-Ah Lee

**Affiliations:** Department of Sociology, Chung-Ang University, Seoul, South Korea

**Keywords:** Digital divide, Structural amplification, Compensation, Bootstrapping, South Korea

## Abstract

**Background and Objectives:**

Despite the growing importance of digital technology for awareness of and access to government services, and the persistent digital divide by education, few studies have investigated the associations among education, digital technology use, and awareness of government services among older adults. This study examined whether and how digital technology use mediates and moderates the relationship between education and service awareness.

**Research Design and Methods:**

Data were obtained from the 2023 National Survey of Older Koreans, a nationally representative survey. Multiple regression analyses were conducted to examine the associations among education, digital technology use, and awareness of government services, and to assess the moderating effect of digital technology use. Bootstrapping was used to examine the mediating effect of digital technology use.

**Results:**

Digital technology use partially mediated the relationship between education and service awareness. Lower educational levels were associated with reduced technology use, which was linked to lower awareness. Digital technology use also had a significant moderating effect. Older adults with lower education and limited digital technology use were the most disadvantaged in terms of service awareness. However, their awareness increased substantially with greater digital technology use, showing a steeper improvement than that of higher-educated counterparts.

**Discussion and Implications:**

A lack of digital technology use may amplify the negative impact of lower education on service awareness. However, less-educated older adults may achieve greater awareness if their digital technology use is high. Promoting digital skills among those with lower education may be crucial for addressing inequalities in service awareness.

Innovation and Translational Significance: Education is associated with awareness of government services among older adults. Although digital technology use is essential for accessing government services, its role in the relationship between education and awareness remains unexplored. This study showed that older adults with lower education had lower digital technology use, leading to reduced awareness of government services. However, those with lower education showed markedly increased awareness as their digital technology use increased. To mitigate disparities in service awareness among older adults, policy efforts should prioritize accessible and age-friendly digital education programs tailored to the needs of older adults, particularly those with lower education.

Amid population aging, numerous countries are actively endeavoring to disseminate information about government services to older adult citizens. This effort stems from the recognition that awareness of government services is a crucial factor in ensuring access to and utilization of the services (Tindale et al., 2011; Xu et al., 2022). Equal awareness of government services may ameliorate disparities not only in service utilization but also in health among older adults. Therefore, understanding the mechanisms that shape service awareness is essential for improving accessibility and promoting equity in public services and health.

Not surprisingly, awareness of services is not observed equitably across social groups but is influenced by social factors such as income and education (Chen et al., 2013; Tindale et al., 2011). Particularly, education can be a critical factor in shaping disparities in service awareness among older adults (e.g., Chen et al., 2013). Individuals with higher education have greater awareness of public services than their less-educated counterparts (Chen et al., 2013; Joseph et al., 2015; Strain & Blandford, 2002), potentially because of their greater cognitive capability, health literacy, and economic status (Cutler & Lleras-Muney, 2010; van der Heide et al., 2013). Given that the influence of education accumulates over the life course (Mirowsky & Ross, 2005), education may deepen the disparity in information and literacy related to government services among older adults.

Despite the empirical findings and contributions of previous studies, our understanding of government service awareness among older adults remains limited. First, few studies have explored the mechanisms through which education influences older adults’ awareness of government services. Investigating the pathways between education and service awareness is essential for mitigating the negative impact of lower education on awareness of government services. Identifying a key mediator amenable to social intervention could provide practical implications for reducing disparities in awareness of government services by educational level. Moreover, despite the widespread digitalization of government services and the persistent digital divide among older adults, little research has investigated the associations between education and older adults’ awareness of government services in relation to their digital technology use. Digital technology use may be a key determinant of service awareness in contemporary societies that increasingly rely on digital platforms to distribute information on public services (United Nations, 2020). People obtain information about social services from government websites and access them online. This trend may lead to the exclusion of older adults with insufficient digital skills from these services. In addition, digital technology use is significantly affected by the educational level of older adults (Friemel, 2016; Hargittai et al., 2019). Thus, it is prudent to examine whether digital technology use is a significant mediator of the relationship between education and awareness of government services.

Beyond potential mediation, digital technology use may also moderate the relationship between education and service awareness. A couple of studies on the mental well-being of older adults have shown that digital technology use is more beneficial for disadvantaged older adults in terms of social interactions (Lee et al., 2021) and physical limitations (Fang et al., 2018). These studies imply that digital technology use can be a useful resource for ameliorating the deleterious effects of lower education on older adults. To the author’s knowledge, however, no study has examined the mediating and moderating effects of digital technology use in the association between education and awareness of government services among older adults using an integrative approach.

To investigate the role of digital technology use, this study focused on Korean older adults aged 65 years and older using a nationally representative sample. South Korea (hereafter Korea) is a rapidly aging country. The percentage of older adults in Korea’s total population was 12.2% in 2013, 19.0% in 2023, and 20% in 2024 (Statistics Korea, 2025). Given the rapid pace of population aging, the Korean government has established various policies and welfare services for older adults, including dementia screening and support programs, customized care services, and a long-term care insurance system (Ministry of Health and Welfare, 2025). Furthermore, Korea is a leading country in terms of governmental digitalization (Turner et al., 2022). Specifically, in 2017, the Korean government consolidated several fragmented e-government systems into a single platform, *Government 24*. This integrated service not only provides access to policy information but also enables citizens to explore customized benefits based on individual or household characteristics. In 2022, the government declared its vision for the Digital Platform Government and strengthened the digitalization of public services (Ministry of the Interior and Safety, 2022). Consequently, Korea’s e-government services were ranked fourth worldwide (United Nations, 2024).

Access to digital devices is also relatively high, even among older adults in Korea. As of 2023, 93.3% of Koreans in their 60s and 63.9% of those aged 70 and older used the internet. Smartphone ownership was also high, with 97.2% of those in their 60s and 79% of those aged 70 and older reporting ownership (Ministry of Science and ICT & National Information Society Agency, 2023). However, their digital skills remain lower than those of the general population. Digital technology capability (with the general population indexed at 100), assessed by the ability to use PCs and mobile devices, was 60.9 among those in their 60s and 30.2 among those aged 70 and older (Ministry of Science and ICT & National Information Society Agency, 2023). Therefore, analyzing the Korean case would provide valuable insights that may be applicable to other countries amid global digitalization.

In sum, this study aimed to investigate the associations among educational level, digital technology use, and awareness of government services among older adults aged 65 and older. As an integrated approach, this study examined whether and how digital technology use mediates and moderates the relationship between education and service awareness. By exploring these dual effects, this study provides empirical evidence and practical implications for reducing disparities in service awareness among older adults, given the growing importance of digital technology use.

## Background

### Education as a foundational resource

Education, operating as human capital, benefits various dimensions, including socioeconomic conditions, individuals’ life trajectories, and health in later life (Ericsson et al., 2023; Mirowsky & Ross, 2005). Higher education is also associated with greater awareness of public services among older adults (e.g., Chen et al., 2013; Joseph et al., 2015), although some studies have reported nonsignificant effects of education (Hopp et al., 2025; Tindale et al., 2011). Those with higher education demonstrated greater awareness of free preventive care (Chen et al., 2013), government facilities (Joseph et al., 2015), and e-government services (Hong & Choi, 2020). Caregivers’ education is also important. Caregivers with lower education reported lower awareness of services available to older adults that they care for (Strain & Blandford, 2002), thereby leading to lower service utilization.

The positive association between education and service awareness can be explained from theoretical perspectives and empirical findings that emphasize education as a critical resource acquired in early life stages, which leads to other resources or advantages over the life course. Life course perspectives, including cumulative advantage/disadvantage theory (Dannefer, 2003; O’Rand, 2003) and cumulative inequality theory (Ferraro & Shippee, 2009), posit that education is an initial factor affecting diverse life conditions and resources that accumulate over the life course. Higher education in early adulthood can lead to not only higher socioeconomic status but also other advantages. Specifically, higher education leads to larger social networks (Ajrouch et al., 2005), greater social support (Van Groenou & Van Tilburg, 2003), and greater health literacy among older adults (van der Heide et al., 2013), all of which may increase exposure to information on public services. Furthermore, higher education is associated with greater cognitive function across the life span (Lövdén et al., 2020), enabling individuals to interpret and navigate complex information more effectively. Similarly, a study reported that higher education was associated with more positive health behaviors, partly because those with higher education had greater economic conditions, health-related knowledge, and cognitive abilities (Cutler & Lleras-Muney, 2010). Consequently, these findings suggest that education functions as a foundational resource that contributes to multiple advantages that can enhance awareness of government services.

### The potential roles of digital technology use

With the development of digital technology, digital governments have emerged and expanded worldwide. A key challenge and goal of government digitalization is to provide accountable and inclusive digital services for all (United Nations, 2020). However, awareness of and access to services are not equally distributed due to the digital divide (Hong & Choi, 2020; Olsson & Viscovi, 2023). Given that older adults generally exhibit lower digital skills and e-health literacy compared to younger generations (Ministry of Science and ICT & National Information Society Agency, 2023; Tennant et al., 2015), digital technology use may be more critical to the disparity in awareness of government services among older adults.

A few studies have shown that education is positively associated with digital technology use (Friemel, 2016; Hargittai et al., 2019; Tennant et al., 2015). Those with higher education are likely to have greater digital utilization capabilities than their counterparts. Furthermore, educational disparity in digital technology use might be more pronounced among older adults, given that they are digital immigrants rather than natives (Prensky, 2001). Digital natives process information more quickly and are better at multitasking than digital immigrants (Prensky, 2001), suggesting that they possess cognitive aspects more suited to learning and efficiently using digital technology. In contrast, older adults were not exposed to digital technology when they were young, leading to higher cognitive and psychological barriers to learning about digital technology.

Specifically, compared to younger adults, older adults generally have lower working memory and slower cognitive processing speed, despite some improvements through practice (Brigman & Cherry, 2002). They also face greater psychological barriers, such as anxiety, fear, and resistance to digital technology, referred to as *technophobia* (Smrke et al., 2025). Cognitive function and psychological barriers within this group also vary by education. Older adults with higher education demonstrate better working memory and performance (de Souza-Talarico et al., 2007), which can facilitate learning and adopting digital skills. Lower-educated older adults tend to experience greater fear and discomfort with digital technology than their highly educated counterparts (Nimrod, 2018; Smrke et al., 2025). Furthermore, prior experience with digital technology increases confidence and openness to learning and using it, even within the older adult group (Lin et al., 2025). This suggests that their lower levels of exposure to digital technology, compared to digital natives, may lead to lower motivation and greater psychological resistance to adopting digital technology. Consequently, digital technology use may serve as a mediator in the relationship between education and awareness of government services. Older adults with lower education are likely to use digital technology less, which in turn may reduce their awareness of government services.

Alternatively, digital technology use could moderate the relationship between education and awareness of government services, as it is more beneficial to older adults with fewer social interactions (Lee et al., 2021) and greater physical limitations (Fang et al., 2018). Given that digital skills are an important capability for accessing information in contemporary society, digital technology use can play a significant role in alleviating the disadvantages associated with lower education among older adults. In addition, although less-educated older adults generally have lower digital skills and cognitive function (Lövdén et al., 2020; Ministry of Science and ICT & National Information Society Agency, 2023), their digital skills can be improved by practice or training (Miller et al., 2024; Pihlainen et al., 2021), and improvements in digital technology lead to slower declines in processing speed and general cognitive function (Li et al., 2024). Considering that digital technology use may not be solely determined by education, less-educated older adults may have increased awareness of government services if they possess high digital skills.

The potential moderation of digital technology use may be discussed from theoretical perspectives such as resource substitution (Ross & Mirowsky, 2006, 2011) and compensatory leveling (Schafer et al., 2013). According to these perspectives, the beneficial effect of a given resource is greater for those who lack other resources. From this viewpoint, education is a critical resource that ameliorates the negative effects of certain disadvantages such as childhood misfortune and family background (Ross & Mirowsky, 2011; Schafer et al., 2013). Similarly, digital technology use by older adults can also be considered an important resource that mitigates the disadvantages of low education. Less-educated people may rely more on digital technology to increase their awareness of government services. Thus, digital technology use may serve as an alternative resource that compensates for the negative effect of lower education on awareness of government services. Accordingly, we hypothesize that older adults with lower education will have improved levels of service awareness if they have greater digital technology use.

## Method

### Data

Data were drawn from the 2023 National Survey of Older Koreans, a nationally representative survey conducted by the Korea Institute for Health and Social Affairs. The target population was noninstitutionalized Korean older adults aged 65 and older (Kang et al., 2023). The survey was conducted using a stratified two-stage cluster sampling method with districts as the primary sampling units and households as the secondary sampling units. A total of 7,605 households were selected from 977 districts, and older adults living in these households were surveyed (Kang et al., 2023). The survey was conducted from September 4 to November 12, 2023. In our study, 9,951 participants were analyzed after excluding proxy reports (*n *= 127).

### Measures

#### Dependent variable

Awareness of government services was measured by asking whether respondents are aware of each of eight government services or institutions for older adults: (1) Customized Care Service; (2) Long-term Care Insurance; (3) Emergency Safety and Reassurance Service for older adults living alone and individuals with disabilities; (4) Early Dementia Screening Program; (5) Dementia Treatment and Management Expense Support Program; (6) Dementia Safety Center; (7) Hospice and Palliative Care Program; (8) Older Adult Protection Institutions (Abuse prevention and response institutions). The response categories for each item were: 1 (I do not know), 2 (I have heard of it, but I am not familiar with the details), and 3 (I am well aware of it). The sum of all eight items was used as the dependent variable, with a higher score indicating a greater level of awareness of government services. The reliability score of the eight items was 0.873 (Cronbach’s α).

#### Educational level

Educational level was categorized into four groups: (1) no schooling completed; (2) elementary school education; (3) middle school education; and (4) high school education or higher. The reference group for the analytical models was no schooling.

#### Digital technology use as potential mediator and moderator

Digital technology use was measured by asking the question, “Do you perform the following activities on a PC, smartphone, tablet, or electronic kiosk without anyone’s assistance?” Respondents were asked about 13 digital activities: (1) receiving text messages; (2) sending text messages; (3) video calling; (4) searching for information such as news and weather; (5) taking photos and video recording; (6) listening to music (MP3); (7) playing games; (8) watching videos (movies, TV programs, etc.); (9) using social networking services; (10) online commerce (online shopping, reservations, ordering food); (11) online transactions (internet banking, stock trading, etc.); (12) searching for and installing applications; and (13) placing orders and making appointments using kiosks in restaurants, hospitals, etc. Digital technology use was estimated by totaling the number of digital activities that the respondents utilize. Higher values indicated more extensive task performance using digital technology.

#### Covariates

Sociodemographic factors included age, gender (female = 1), marital status, household income, and homeownership status. Age was measured in years, and marital status was categorized into four groups: (1) married; (2) widowed; (3) divorced or separated; and (4) never married. The reference group was married individuals. Household income was measured in quintiles. Home ownership was measured as a binary variable (owning a house = 1 vs. renting or other = 0). Health status was also included in the analyses based on (1) cognitive function, (2) chronic condition, and (3) self-rated health. Cognitive function was measured using the Korean version of the Mini-Mental State Examination for Dementia Screening (MMSE-DS), which consists of 30 questions. The total MMSE-DS score (i.e., the number of correct answers) was included in the analytic models. Chronic condition was a dummy variable, where respondents diagnosed by a doctor with at least one chronic health condition were assigned a value of 1. Self-rated health scores ranged from 1 (very unhealthy) to 5 (very healthy).

Two additional covariates (i.e., area of residence and digital accessibility) were included as potential confounders that could be associated with digital technology use and awareness of government services. The area of residence was measured as a dummy variable indicating whether respondents lived in a rural or urban area (rural = 1). Digital accessibility was assessed with four items: (1) whether the household had internet access and whether the respondent owned the following items: (2) a smartphone, (3) a computer (desktop, laptop, or tablet PC), and (4) a smartwatch. Each affirmative response was coded as 1, and the scores were summed to construct the variable, ranging from 0 to 4. Higher values indicated greater accessibility. There were no missing cases in our variables.

### Analytical strategy

Multiple regression analyses were used to estimate the associations between education, digital technology use, and awareness of government services. Three analytical models were conducted. Model 1 included educational level with covariates only, whereas Model 2 added digital technology use. For the moderation test, Model 3 included the interaction terms between educational level and digital technology use.

Bootstrapping was used to examine the mediating effects of digital technology use on the relationship between education and awareness of government services (Hayes & Preacher, 2014). Although the causal steps approach proposed by Baron and Kenny (1986) has been widely used, it has been criticized for its low statistical power and the lack of quantification of indirect effects (Hayes, 2009). The bootstrapping technique is also advantageous over the *Sobel* test, as it provides greater statistical power and does not require the normality assumption in the sampling distribution of the indirect effect (Hayes, 2009; Ng & Lin, 2016).

Thus, bootstrapping with 5,000 resamples was conducted as a formal test of mediation. During the bootstrapping procedure, all covariates included in the multiple regression analyses were controlled for both the mediator (i.e., digital technology use) and the outcome variable (i.e., awareness of government services). As preliminary analyses, Models 1 and 2 from the multiple regression analyses were presented in accordance with Baron and Kenny (1986)’s causal steps approach. The analytical results of the multiple regression and bootstrapping were weighted. The weights were created by the Korean Institute for Health and Social Affairs to reflect the sampling methods, nonresponse rates, and distribution of the older adult population (Kang et al., 2023).

## Results

### Sample characteristics

Table 1 presents the descriptive statistics of the variables for all respondents and for each education group. ANOVA or chi-square tests were conducted to examine whether the means or distributions of variables significantly differed across the four education groups. The mean score for the dependent variable, awareness of government services, was 15.833, with a range of 8 to 24. Its skewness and kurtosis were −0.043 and 2.122, respectively (not shown in Table 1), which do not violate normality. Among the four education groups, those who had not completed any formal education (no schooling) had the lowest average score for awareness of government services (13.854) compared to those with elementary school (15.320), middle school (16.227), and high school education or higher (16.839). The ANOVA results for the dependent variable revealed that the mean awareness scores differed statistically across the four education groups.

**Table 1.** Descriptive statistics and bivariate analyses.

Variable	Total (*N *= 9,951)			No schooling (*n *= 1,435)		Elementary school (*n *= 2,920)		Middle school (*n *= 2,114)		High school or higher (*n *= 3,482)		ANOVA/chi-square test	
	Mean/Proportion	*SD*	Range	Mean/Proportion	*SD*	Mean/Proportion	*SD*	Mean/Proportion	*SD*	Mean/Proportion	*SD*		
**Awareness of government services**	15.833	4.094	8–24	13.854	4.048	15.320	4.073	16.227	3.997	16.839	3.822	14.976	**
**Educational level**													
**No schooling**	0.144		0.1										
**Elementary school**	0.293		0.1										
**Middle school**	0.212		0.1										
**High school or higher**	0.350		0.1										
**Age**	74.024	6.751	65–103	80.433	6.038	76.025	6.249	72.462	5.541	70.653	5.544	60.638	***
**Gender (female = 1)**	0.616		0.1	0.838		0.697		0.581		0.477		671.802	***
**Marital status**												1200.000	***
**Married**	0.591		0.1	0.321		0.531		0.644		0.721			
**Widowed**	0.328		0.1	0.646		0.409		0.257		0.172			
**Divorced or separated**	0.074		0.1	0.026		0.056		0.088		0.099			
**Never married**	0.007		0.1	0.006		0.004		0.010		0.008			
**Household income**	2.901	1.395	1–5	2.046	1.140	2.510	1.242	2.977	1.334	3.534	1.349	67.671	***
**Home ownership**	0.803		0.1	0.782		0.809		0.779		0.823		21.213	***
**Cognitive function**	24.503	4.766	0–30	19.321	4.957	23.933	3.952	25.573	3.453	26.469	4.309	252.762	***
**Chronic condition**	0.863		0.1	0.939		0.908		0.875		0.787		294.514	***
**Self-rated health**	3.168	0.886	1–5	2.601	0.885	2.983	0.849	3.230	0.830	3.520	0.781	39.935	***
**Area of residence (rural = 1)**	0.299		0.1	0.527		0.383		0.240		0.171		762.015	***
**Digital accessibility**	1.381	1.035	0–4	0.484	0.697	1.039	0.864	1.454	0.910	1.994	0.961	197.295	***
**Digital technology use**	4.324	3.546	0–13	1.054	1.608	2.704	2.485	4.667	3.000	6.821	3.366	990.395	***

*Note*. *SD*s (standard deviations) of binary variables are not presented. Unweighted statistics are presented.

* *p *< .05.

** *p *< .01.

*** *p *< .001.

For other variables, the means or distributions differed significantly across the education groups. The respondents’ mean age was the highest in the lowest education group. Women were more likely to belong to the lowest education group. Other sociodemographic variables also varied according to educational level. Regarding health status, the means of cognitive function and self-rated health were higher in the highest education group. Approximately 93.9% of respondents in the lowest education group (no schooling) had at least one chronic health condition. A higher proportion of respondents with lower education resided in rural areas. Mean scores of digital accessibility also differed across educational levels. The level of digital accessibility was highest among those with a high school education or higher. The mean scores for digital accessibility were 0.484 and 1.994 for the lowest and highest education groups, respectively.

The level of digital technology use was highest among those with a high school education or higher. The mean scores for digital technology use from the lowest to highest education groups were 1.054, 2.704, 4.667, and 6.821, respectively. As supplementary analyses, Bonferroni mean comparison tests across the four education groups were conducted for awareness of government services and digital technology use. All pairwise comparisons were statistically significant. Higher education groups had higher mean scores for service awareness and digital technology use than lower education groups (see Supplementary Table 1), suggesting that educational gradients in service awareness and digital technology use exist.

### Educational level, digital technology use, and awareness of government services

Table 2 presents the results of multiple regression analyses examining the associations between education, digital technology use, and awareness of government services among older adults. In Model 1, all three educational variables were significantly associated with awareness of government services. Those with elementary, middle, and high school education or higher had greater levels of awareness than those without formal education. Education was still significantly associated with awareness of government services in Model 2, where digital technology use was added, although its coefficients were reduced. Digital technology use was also significantly associated with awareness of government services. As the level of digital technology use increased, so did the level of awareness.

**Table 2.** Multiple regression analyses examining associations between educational level, digital technology use, and awareness of government services among older adults.

Variable	Model 1		Model 2		Model 3	
**Educational level (no schooling = referent)**						
**Elementary school**	0.777	***	0.764	***	0.910	***
	(0.177)		(0.177)		(0.228)	
**Middle school**	1.173	***	1.035	***	1.169	***
	(0.194)		(0.194)		(0.269)	
**High school or higher**	1.129	***	0.816	***	1.412	***
	(0.199)		(0.201)		(0.266)	
**Age**	0.035	***	0.054	***	0.056	***
	(0.009)		(0.010)		(0.010)	
**Gender (female = 1)**	0.189	^+^	0.206	*	0.218	*
	(0.099)		(0.099)		(0.098)	
**Marital status (married = referent)**						
**Widowed**	0.206	^+^	0.190	^+^	0.195	^+^
	(0.115)		(0.114)		(0.114)	
**Divorced or separated**	0.228		0.187		0.189	
	(0.180)		(0.179)		(0.178)	
**Never married**	−0.356		−0.293		−0.311	
	(0.533)		(0.523)		(0.524)	
**Household income**	−0.064	^+^	−0.075	^+^	−0.072	^+^
	(0.038)		(0.038)		(0.038)	
**Home ownership**	0.074		0.090		0.090	
	(0.122)		(0.122)		(0.121)	
**Cognitive function**	0.100	***	0.094	***	0.091	***
	(0.010)		(0.010)		(0.010)	
**Chronic condition**	−0.297	*	−0.257	^+^	−0.271	*
	(0.138)		(0.137)		(0.136)	
**Self-rated health**	0.267	***	0.201	**	0.198	**
	(0.059)		(0.060)		(0.060)	
**Area of residence (rural = 1)**	−1.047	***	−1.059	***	−1.046	***
	(0.102)		(0.102)		(0.102)	
**Digital accessibility**	0.650	***	0.373	***	0.362	***
	(0.054)		(0.062)		(0.062)	
**Digital technology use (DTU)**			0.181	***	0.395	***
			(0.020)		(0.079)	
**Interaction terms**						
**Elementary school × DTU**					−0.171	*
					(0.084)	
**Middle school × DTU**					−0.182	*
					(0.083)	
**High school or higher × DTU**					−0.256	**
					(0.080)	
**Constant**	8.955	***	7.618	***	7.283	***
	(0.885)		(0.902)		(0.912)	
***N***	9.951		9.951		9.951	
***F***	67.67	***	68.85	***	58.52	***
***R* ^2^**	0.108		0.117		0.119	

*Note.* Unstandardized coefficients are presented. The numbers in parentheses are robust standard errors. The statistics were weighted.

+ *p *< .10.

* *p *< .05.

** *p *< 0.1.

*** *p *< .001.

Regarding other covariates, age, gender, and health status were significantly associated with awareness of government services across all models. Women had greater awareness than men. Age, cognitive function, and self-rated health were positively associated with awareness of government services. As cognitive function and self-rated health increased, the level of awareness of government services also increased. Chronic condition was marginally negatively associated with service awareness in Model 2. Respondents living in rural areas had lower levels of awareness of government services than those living in urban areas. Digital accessibility was positively associated with service awareness. As the level of digital accessibility increased, the level of awareness increased.

**Figure 1.**
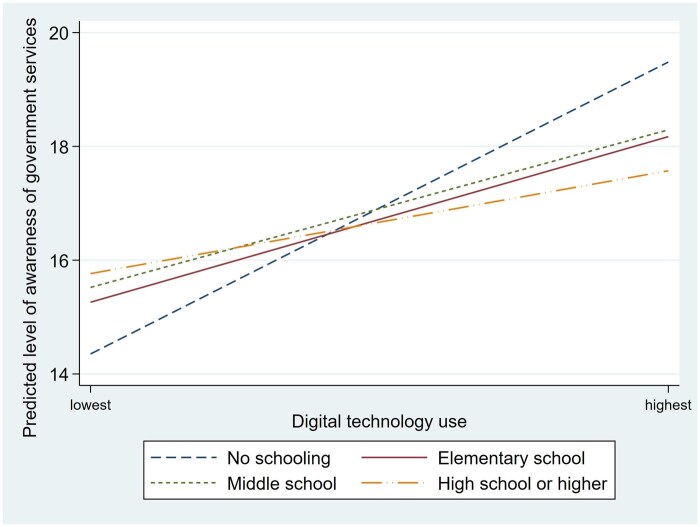
Predicted levels of awareness of government services by educational level and digital technology use.

To test moderation, Model 3 in Table 2 included three interaction terms between education and digital technology use. All interaction terms were statistically significant. This means that the association between education and awareness of government services varied with the levels of digital technology use. Figure 1 shows the interactions between education and digital technology use for awareness of government services, as estimated in Model 3. The Y-axis indicates the predicted level of awareness of government services among older adults. Figure 1 shows that awareness of government services increased with the level of digital technology use in all four groups. However, the slope reflecting this increase was much steeper for the lowest education group. Specifically, those without formal education had much lower awareness of government services when they had lower digital technology use. In contrast, their awareness of government services substantially increased as their digital technology use levels increased.

Table 3 presents the bootstrapping results examining the indirect effects of education via digital technology use (i.e., the mediating effects of digital technology use). The direct effects of education on awareness of government services were also estimated. Given that the independent variable (i.e., education) was multi-categorical, the indirect and direct effects represent quantified estimates of the effect of each education group relative to the referent group (i.e., those without formal education) (Hayes & Preacher, 2014). As shown in Table 3, digital technology use significantly mediated the relationship between educational level and awareness of government services.

**Table 3.** Indirect and direct effects of educational level examined by Bootstrapping.

Variable	Coefficient		95% Confidence interval	
	(Standard errors)		Lower bound	Upper bound
**Indirect effects via digital technology use**				
**Elementary school education**	0.013		−0.012	0.039
	(0.013)			
**Middle school education**	0.138	***	0.095	0.182
	(0.022)			
**High school education or higher**	0.312	***	0.235	0.389
	(0.039)			
**Total indirect effect**	0.464	***	0.335	0.592
	(0.066)			
**Direct effects**				
**Elementary school education**	0.764	***	0.423	1.106
	(0.174)			
**Middle school education**	1.035	***	0.655	1.415
	(0.194)			
**High school education or higher**	0.816	***	0.423	1.210
	(0.201)			
**Total direct effect**	2.615	***	1.593	3.637
	(0.521)			

*Note.* The numbers in parentheses are robust standard errors. The statistics are weighted.

*** *p *< .001.

The indirect effects of middle and high school education or higher were statistically significant (*b* = 0.138 for middle school education and *b* = 0.312 for high school education or higher). Compared with those without formal education, respondents with middle school and high school education or higher had 0.138 and 0.312 units higher awareness of government services, respectively, as a result of the positive effect of digital technology use. In contrast, the indirect effect of elementary school education was not significant, suggesting that the difference in service awareness between those without formal education and those with elementary school education was not substantially explained by differences in digital technology use related to their education. Although the indirect effect of elementary school education was not significant (*b* = 0.013), the results in Table 3 support that digital technology use mediates the effect of education on awareness of government services, given that the indirect effects of two education categories, relative to no schooling, are significantly different from zero (Hayes & Preacher, 2014). The direct effects of education were also significant (*b* = 2.615 for the total direct effect). Adjusting for differences in digital technology use across education groups, those with elementary, middle, and high school education or higher had greater levels of awareness of government services compared with those without formal education.

The relative indirect effect of education through digital technology use, as a ratio relative to its corresponding relative total effect, was 0.151 (= 0.464/[0.464 + 2.615]). This means that approximately 15.1% of the effect of overall education on awareness of government services was mediated by digital technology use. More specifically, compared to those without formal education, the relative indirect effect of middle school education was 0.118 (= 0.138/[0.138 + 1.035]), suggesting that approximately 11.8% of the total effect of middle school education was mediated via digital technology use. For high school education or higher, 27.65% of the total effect was mediated via digital technology use (= [0.464/{0.464 + 0.816}*100]). The higher the educational level, the greater the proportion of the effect that was mediated through digital technology use compared to no schooling.

Two sets of sensitivity analyses were conducted to ensure the robustness of findings. The first set of sensitivity analyses consisted of negative binomial regression analyses of the count outcome variable. The dependent variable, awareness of government services, was initially measured as a continuous variable by summing all eight items asked. Given that the response categories for each item were: 1 (I do not know), 2 (I have heard of it, but I am not familiar with the details), and 3 (I am well aware of it), respondents who selected the second or third category may have been influenced by whether they actually used the services. To account for this, both the second and third categories were coded as 1, indicating awareness of the service, whereas the first category (I do not know) was coded as 0. Then, a count variable was constructed to measure the total number of government services known by each respondent. Using the count variable, negative binomial regression analyses and bootstrapping were conducted and presented in Supplementary Tables 2 and 3, and Supplementary Figure 1. The second set of sensitivity analyses consisted of additional multiple regression analyses and bootstrapping, including years of education as a continuous variable instead of the multi-categorical education variable. The results of the moderation and mediation tests are presented in Supplementary Tables 4 and 5, and Supplementary Figure 2. Both sets of sensitivity analyses largely provided consistent results with the findings.

## Conclusion

The findings showed that education was positively associated with awareness of government services. Older adults with higher education had greater awareness of government services, consistent with previous studies (Chen et al., 2013; Joseph et al., 2015). This can be understood through theoretical perspectives (Dannefer, 2003; Ferraro & Shippee, 2009; O’Rand, 2003) that emphasize education as a critical resource and advantage obtained in early life stages with long-lasting effects over the life course. The positive effect of higher education on awareness of government services may reflect the accumulation of other advantages, including socioeconomic and behavioral aspects (Mirowsky & Ross, 2005).

Interestingly, digital technology use had both mediating and moderating effects on the relationship between education and awareness of government services. The mediation suggests that older adults with lower education are less likely to be aware of government services because they have lower digital technology use. In addition, the moderation indicates that the association between education and awareness of government services varies according to the level of digital technology use. Among older adults with lower education, awareness of government services increased more substantially as digital technology use increased, compared to those with higher education. This can be explained by the resource substitution and compensatory leveling perspectives (Ross & Mirowsky, 2006; Schafer et al., 2013), which emphasize that a particular resource serves as a more beneficial resource to individuals who lack other resources. In this context, high levels of digital technology use may function as a compensatory factor for less-educated older adults.

As both the mediation and moderation of digital technology use were statistically significant, the findings suggest that the negative effects of lower education are exacerbated by suppressed digital technology use, which can be interpreted as *structural amplification* (Hill et al., 2009; Ross et al., 2001). Structural amplification occurs when a factor increases the likelihood of encountering adverse conditions, and the negative effect experienced in such conditions is greater (Mirowsky & Ross, 2005). Older adults with lower education have lower digital technology use, and their lower digital technology use worsens their awareness of government services. Lower digital technology use shaped by lower education can work as a structural amplification factor (Hill et al., 2009; Ross et al., 2001) that broadens the gap among older adults.

Consequently, lower levels of education and digital technology use could be a *double jeopardy* for awareness of government services among older adults. However, digital technology use is an important buffer against the negative consequences of lower education on awareness of government services if older adults achieve high levels of digital technology use. It is important to reduce the digital divide among older adults by educational level to ensure equal awareness of and access to government services in the long run. As cognitive performance can be improved through practice (Brigman & Cherry, 2002) and adopting digital skills may reduce cognitive decline (Li et al., 2024), increasing digital skills among less-educated older adults would be beneficial not only in terms of awareness of government services but also for their cognitive health.

Regarding other covariates, age, gender, and health status were significantly associated with awareness of government services. Women had greater awareness than men, consistent with the findings of a previous study (Hopp et al., 2025). Age, cognitive function, and self-rated health were positively associated with awareness of government services. This finding aligns with a previous study, which reported that those with better self-rated health had greater awareness (Chen et al., 2013). The associations of health conditions with service awareness suggest that older adults in poor health are less likely to be aware of government services, even though they may have greater needs, which calls for targeted policy interventions. In addition, although it is beyond the scope of this study, it is important to consider that health conditions may also mediate and moderate the relationships between education and awareness of government services. Higher-educated older adults generally have better health conditions (Ericsson et al., 2023; Lövdén et al., 2020). At the same time, poorer health conditions influenced by low education may impede the exploration of information on government services.

Some limitations should be addressed. First, although considering education as a preceding factor is valid, causality between digital technology use and awareness of government services cannot be established because the data were cross-sectional. They may have reciprocal relationships that should be investigated using longitudinal data. Second, due to data limitations, this study did not distinguish between types or purposes of digital technology use, nor did it include information on the duration of older adults’ engagement with digital technology. Future research should employ more detailed and nuanced measurements to examine the impact of digital technology use. Finally, education remained significant for awareness of government services after controlling for digital technology use and other covariates. This implies that there might be other factors that mediate educational effects. Further research is needed to investigate other potential mediators.

Despite these limitations, this study uncovered the critical role of digital technology use in the association between education and awareness of government services. Older adults with lower education had lower awareness of government services, partially owing to lower digital technology use. Disparities in digital technology use can amplify the disadvantages faced by older adults with lower education in terms of awareness of government services. However, digital technology use can become an important buffer against the negative consequences of lower education if older adults exhibit high levels of digital technology use. Educating less-educated older adults in digital technology use may help ameliorate disparities in service awareness among older adults. Policy efforts should prioritize accessible and age-friendly digital education programs tailored to the needs of older adults, particularly those with lower education. In addition, alternative communication strategies, such as in-person outreach, printed materials, and public service announcements, should be maintained to ensure equitable access for individuals facing persistent barriers to digital engagement.

## Supplementary Material

igaf146_Supplementary_Data

## Data Availability

Data are publicly available from the Microdata Integrated Service of Statistics Korea (https://mdis.kostat.go.kr). This study was not preregistered.
