# Supplementary material for: Associations between education and awareness of government services among older adults: an integrated approach to examine the role of digital technology use
Source: Innov Aging. 2025 Dec 15;10(4):igaf146. doi: 10.1093/geroni/igaf146 (PMC12995426; doi:10.1093/geroni/igaf146)
Supplement: igaf146_Supplementary_Data [file igaf146_supplementary_data.zip › igaf146_Supplementary_Data.zip!/innage suppl Lee MA.docx]

***Innovation in Aging* Supplementary Material: Lee. Associations between Education and Awareness of Government Services among Older Adults: An Integrated Approach to Examine the Role of Digital Technology Use.**

Supplementary Table 1. Bonferroni tests for pairwise mean comparisons of awareness of government services and digital technology use across four education groups.

|  | Awareness of government services | | | | Digital technology use | | | |
| --- | --- | --- | --- | --- | --- | --- | --- | --- |
|  | Contrast | | 95% Confidence Interval | | Contrast | | 95% Confidence Interval | |
|  | (SE) | | lower | upper | (SE) | | lower | upper |
| Elementary school vs. No schooling | 1.466 | *** | 1.129 | 1.804 | 1.650 | *** | 1.409 | 1.892 |
|  | (0.128) |  |  |  | (0.092) |  |  |  |
| Middle school vs. No schooling | 2.373 | *** | 2.015 | 2.731 | 3.613 | *** | 3.356 | 3.869 |
|  | (0.136) |  |  |  | (0.097) |  |  |  |
| High school or higher vs. No schooling | 2.985 | *** | 2.657 | 3.314 | 5.766 | *** | 5.531 | 6.001 |
|  | (0.124) |  |  |  | (0.089) |  |  |  |
| Middle school vs. Elementary school | 0.907 | *** | 0.608 | 1.206 | 1.963 | *** | 1.749 | 2.176 |
|  | (0.113) |  |  |  | (0.081) |  |  |  |
| High school or higher vs. Elementary school | 1.519 | *** | 1.256 | 1.782 | 4.116 | *** | 3.928 | 4.304 |
|  | (0.100) |  |  |  | (0.071) |  |  |  |
| High school or higher vs. Middle school | 0.612 | *** | 0.323 | 0.900 | 2.154 | *** | 1.947 | 2.360 |
|  | (0.109) |  |  |  | (0.078) |  |  |  |

***p<0.001

Supplementary Table 2. Negative Binomial Regression of the count outcome

|  | Model 1 | | Model 2 | | Model 3 | |
| --- | --- | --- | --- | --- | --- | --- |
| Educational level (no schooling=referent) |  |  |  |  |  |  |
| Elementary school | 0.104 | *** | 0.103 | *** | 0.134 | *** |
|  | (0.021) |  | (0.021) |  | (0.028) |  |
| Middle school | 0.136 | *** | 0.127 | *** | 0.174 | *** |
|  | (0.022) |  | (0.022) |  | (0.031) |  |
| High school or higher | 0.134 | *** | 0.113 | *** | 0.211 | *** |
|  | (0.022) |  | (0.022) |  | (0.029) |  |
| Age | 0.001 |  | 0.002 | ** | 0.003 | ** |
|  | (0.001) |  | (0.001) |  | (0.001) |  |
| Gender (female=1) | 0.007 |  | 0.008 |  | 0.010 |  |
|  | (0.009) |  | (0.009) |  | (0.009) |  |
| Marital status (married=referent) |  |  |  |  |  |  |
| Widowed | 0.012 |  | 0.011 |  | 0.011 |  |
|  | (0.011) |  | (0.011) |  | (0.011) |  |
| Divorced or separated | 0.027 | + | 0.024 |  | 0.024 |  |
|  | (0.015) |  | (0.015) |  | (0.015) |  |
| Never married | -0.071 |  | -0.066 |  | -0.069 |  |
|  | (0.057) |  | (0.056) |  | (0.056) |  |
| Household income | -0.015 | *** | -0.016 | *** | -0.016 | *** |
|  | (0.004) |  | (0.004) |  | (0.003) |  |
| Home ownership | 0.021 | + | 0.022 | + | 0.022 | + |
|  | (0.012) |  | (0.012) |  | (0.012) |  |
| Cognitive function | 0.012 | *** | 0.011 | *** | 0.011 | *** |
|  | (0.001) |  | (0.001) |  | (0.001) |  |
| Chronic condition | -0.013 |  | -0.010 |  | -0.012 |  |
|  | (0.012) |  | (0.012) |  | (0.012) |  |
| Self-rated health | 0.027 | *** | 0.022 | *** | 0.021 | *** |
|  | (0.006) |  | (0.006) |  | (0.006) |  |
| Area of residence (rural=1) | -0.112 | *** | -0.112 | *** | -0.110 | *** |
|  | (0.011) |  | (0.011) |  | (0.011) |  |
| Digital accessibility | 0.053 | *** | 0.034 | *** | 0.032 | *** |
|  | (0.005) |  | (0.005) |  | (0.005) |  |
| Digital technology use (DTU) |  |  | 0.012 | *** | 0.051 | *** |
|  |  |  | (0.002) |  | (0.007) |  |
| Interaction terms |  |  |  |  |  |  |
| Elementary school*DTU |  |  |  |  | -0.031 | *** |
|  |  |  |  |  | (0.008) |  |
| Middle school*DTU |  |  |  |  | -0.037 | *** |
|  |  |  |  |  | (0.007) |  |
| High school or higher*DTU |  |  |  |  | -0.044 | *** |
|  |  |  |  |  | (0.007) |  |
| Constant | 1.193 | *** | 1.099 | *** | 1.036 | *** |
|  | (0.082) |  | (0.084) |  | (0.085) |  |
| N | 9,951 |  | 9,951 |  | 9,951 |  |
| Chi^2^ | 849.06 | *** | 924.58 | *** | 916.06 | *** |
| Pseudo R^2^ | 0.024 |  | 0.025 |  | 0.026 |  |

Notes: Unstandardized coefficients are presented. The numbers in parentheses are robust standard errors. The statistics were weighted.

+*p*<0.1, **p*<0.05, ***p*<0.01, ****p*<0.001

Supplementary Table 3. Indirect and direct effects of educational level on the count outcome, estimated by Bootstrapping

|  | Coefficients | | 95% Confidence interval | |
| --- | --- | --- | --- | --- |
|  | (Standard errors) | | Lower bound | Upper bound |
| Indirect effects via digital technology use |  |  |  |  |
| Elementary school education | 0.001 |  | -0.001 | 0.003 |
|  | (0.001) |  |  |  |
| Middle school education | 0.010 | *** | 0.006 | 0.013 |
|  | (0.002) |  |  |  |
| High school education or higher | 0.022 | *** | 0.015 | 0.028 |
|  | (0.003) |  |  |  |
| Total indirect effect | 0.032 | *** | 0.022 | 0.042 |
|  | (0.005) |  |  |  |
| Direct effects |  |  |  |  |
| Elementary school education | 0.103 | *** | 0.063 | 0.143 |
|  | (0.020) |  |  |  |
| Middle school education | 0.127 | *** | 0.084 | 0.170 |
|  | (0.022) |  |  |  |
| High school education or higher | 0.113 | *** | 0.070 | 0.156 |
|  | (0.022) |  |  |  |
| Total direct effect | 0.343 | *** | 0.224 | 0.462 |
|  | (0.061) |  |  |  |

Notes: The numbers in parentheses are robust standard errors. The statistics are weighted.

****p*<0.001

Supplementary Table 4. Multiple regression analyses of awareness of government services, including years of education

|  | Model 1 |  | Model 2 | | Model 3 | |
| --- | --- | --- | --- | --- | --- | --- |
| Years of education | 0.088 | *** | 0.061 | *** | 0.122 | *** |
|  | (0.015) |  | (0.015) |  | (0.020) |  |
| Age | 0.033 | *** | 0.052 | *** | 0.056 | *** |
|  | (0.009) |  | (0.010) |  | (0.010) |  |
| Gender (female=1) | 0.215 | * | 0.236 | * | 0.236 | * |
|  | (0.100) |  | (0.099) |  | (0.099) |  |
| Marital status (married=referent) |  |  |  |  |  |  |
| Widowed | 0.188 |  | 0.169 |  | 0.197 | + |
|  | (0.115) |  | (0.114) |  | (0.114) |  |
| Divorced or separated | 0.202 |  | 0.163 |  | 0.182 |  |
|  | (0.181) |  | (0.180) |  | (0.179) |  |
| Never married | -0.401 |  | -0.348 |  | -0.321 |  |
|  | (0.533) |  | (0.523) |  | (0.524) |  |
| Household income | -0.074 | + | -0.087 | * | -0.081 | * |
|  | (0.038) |  | (0.038) |  | (0.038) |  |
| Home ownership | 0.055 |  | 0.073 |  | 0.092 |  |
|  | (0.122) |  | (0.122) |  | (0.122) |  |
| Cognitive function | 0.104 | *** | 0.100 | *** | 0.092 | *** |
|  | (0.010) |  | (0.010) |  | (0.010) |  |
| Chronic condition | -0.262 | + | -0.219 |  | -0.254 | + |
|  | (0.138) |  | (0.137) |  | (0.137) |  |
| Self-rated health | 0.265 | *** | 0.202 | ** | 0.195 | ** |
|  | (0.059) |  | (0.060) |  | (0.060) |  |
| Area of residence (rural=1) | -1.043 | *** | -1.050 | *** | -1.034 | *** |
|  | (0.102) |  | (0.102) |  | (0.101) |  |
| Digital accessibility | 0.623 | *** | 0.364 | *** | 0.357 | *** |
|  | (0.054) |  | (0.062) |  | (0.062) |  |
| Digital technology use (DTU) |  |  | 0.168 | *** | 0.347 | *** |
|  |  |  | (0.020) |  | (0.042) |  |
| Interaction term |  |  |  |  |  |  |
| Years of education*DTU |  |  |  |  | -0.017 | *** |
|  |  |  |  |  | (0.004) |  |
| Constant | 9.181 | *** | 7.909 | *** | 7.266 | *** |
|  | (0.878) |  | (0.893) |  | (0.909) |  |
| N | 9,951 |  | 9,951 |  | 9,951 |  |
| F | 77.32 |  | 77.57 |  | 73.55 |  |
| R^2^ | 0.107 |  | 0.1151 |  | 0.1179 |  |

Notes: Unstandardized coefficients are presented. The numbers in parentheses are robust standard errors. The statistics were weighted.

+*p*<0.1, **p*<0.05, ***p*<0.01, ****p*<0.001

Supplementary Table 5. Indirect and direct effects of years of educational on awareness of government services, estimated by Bootstrapping

|  | Coefficients | | 95% Confidence interval | |
| --- | --- | --- | --- | --- |
|  | (Standard errors) | | Lower bound | Upper bound |
| Indirect effect of years of education via digital technology use | 0.027 | *** | 0.020 | 0.033 |
|  | (0.003) |  |  |  |
| Direct effect of years of education | 0.061 | *** | 0.032 | 0.091 |
|  | (0.015) |  |  |  |

Notes: The numbers in parentheses are robust standard errors. The statistics are weighted.

****p*<0.001

Supplementary Figure 1. Predicted levels of the count outcome by educational level and digital technology use, estimated from negative binomial regression

Supplementary Figure 2. Predicted levels of awareness of government services by years of education and digital technology use, estimated from multiple regression
